# Mechanochemically Synthesized ZIF-67/PANI-SSA Composites as Superior Adsorbents for the Removal of Basic Fuchsin from Water

**DOI:** 10.3390/molecules31162871

**Published:** 2026-08-17

**Authors:** Maja Ranković, Danica Bajuk-Bogdanović, Nemanja Gavrilov, Maja Milojević-Rakić, Marjetka Savić, Igor Pašti, Aleksandra Janošević Ležaić, Gordana Ćirić-Marjanović

**Affiliations:** 1 University of Belgrade-Faculty of Physical Chemistry, Studentski trg 12-16, 11158 Belgrade, Serbia; maja.rankovic@ffh.bg.ac.rs (M.R.); danabb@ffh.bg.ac.rs (D.B.-B.); gavrilov@ffh.bg.ac.rs (N.G.); maja@ffh.bg.ac.rs (M.M.-R.); igor@ffh.bg.ac.rs (I.P.); 2 “Vinča” Institute of Nuclear Science, University of Belgrade, National Institute of the Republic of Serbia, 11001 Belgrade, Serbia; metk@vin.bg.ac.rs; 3 Serbian Academy of Sciences and Arts, Kneza Mihaila 35, 11000 Belgrade, Serbia; 4 University of Belgrade-Faculty of Pharmacy, Vojvode Stepe 450, 11221 Belgrade, Serbia; aleksandra.janosevic@pharmacy.bg.ac.rs

**Keywords:** metal–organic framework, ZIF-67, conducting polymer, polyaniline, composite, basic fuchsin, adsorption, environmental protection

## Abstract

In the search for efficient adsorbents for pollutants removal from water, we prepared novel composites of the metal–organic framework ZIF-67 and conducting 1-D nanostructured polyaniline sulfosalicylate (PANI-SSA) in different weight ratios (20–75 wt.% ZIF-67) using a mechanochemical method. Composites were characterized by FAAS, SEM-EDX, TGA, FTIR and Raman spectroscopies, N_2_ sorption, electrical conductivity and zeta-potential measurements and explored as adsorbents for the removal of basic fuchsin (BF) dye from an aqueous medium. TGA revealed improved thermal stability of the composites compared to pure components, while FTIR and Raman indicated strong interactions between PANI-SSA and ZIF-67 causing partial dedoping of the polymer. Composites’ morphology depended on their composition, with 1-D nanostructures observed for lower ZIF-67 contents. All composites are excellent adsorbents for BF, having better adsorption performance than pristine PANI-SSA. The composite with 75 wt.% ZIF-67 exhibited the highest values of maximum adsorption capacity (910 mg g^−1^) and removal efficiency (97.2%), exceeding those for pure ZIF-67. Adsorption processes followed pseudo-second-order kinetics fitting the Langmuir–Freundlich isotherm, indicating monolayer adsorption on a heterogeneous surface. Composite adsorbent can be efficiently regenerated by washing with ethanol and reused. The results highlight the synergistic effects of combining PANI and ZIF-67 into advanced materials for wastewater treatment.

## 1. Introduction

Environmental pollution caused by synthetic dyes discharged from textile, leather, paper, and pharmaceutical industries has become one of the major ecological concerns of the modern era [1,2]. Dyes such as basic fuchsin (BF) and crystal violet are widely applied in industrial processes and laboratory procedures, yet they exhibit low biodegradability, potential mutagenic and carcinogenic effects, and high toxicity to aquatic organisms [3,4,5]. Conventional treatment techniques including ion exchange, electrodialysis, filtration, membrane processes, and advanced oxidation methods, while efficient under controlled conditions, remain economically and practically limited due to high operational costs and generation of toxic by-products [6,7].

A cationic dye basic fuchsin (BF), also called Basic Violet 14, belongs to the triarylmethane class of dyes and is widely used in textile industries for dyeing silk, wool, and cotton [8,9]. It also has anesthetic, bactericidal, and fungicidal properties, and it is used in the medical field in the staining of collagen, muscle, mitochondria, and tubercle bacillus [10]. Due to its toxicity, persistence, carcinogenicity, and non-biodegradability, the release of BF into the environment poses severe risks. Therefore, the removal of BF from wastewater has been extensively studied [5,11,12].

Among the available options, adsorption has emerged as a simple, efficient, and cost-effective method for dye removal from aqueous solutions [13,14]. Activated carbon is considered the most effective adsorbent, but its high price and regeneration difficulties have led to the exploration of alternative low-cost adsorbents [15]. In this context, advanced materials such as metal–organic frameworks (MOFs) and conductive polymers have gained increasing attention for wastewater treatment applications [16,17].

MOFs are a class of crystalline porous materials consisting of metal ions or metal clusters coordinated with organic linkers through coordination bonds [18,19]. While their applications may be limited by low thermal and chemical stability and poor electrical conductivity, MOFs hold significant promise due to their high specific surface area and adjustable porosity, which can be tailored through synthesis conditions and linker selection [20]. Furthermore, the ability to introduce active sites into their structure enables a variety of reactions, making them valuable for applications such as energy conversion and storage, water purification, gas adsorption and separation, and more [21,22].

Zeolitic imidazolate frameworks (ZIFs) are a subclass of MOFs consisting of metal ions such as Zn^2+^, Co^2+^, or Ni^2+^ or clusters coordinated with organic ligands in a three-dimensional framework [23,24]. These materials’ unique properties, such as high crystallinity and porosity [25], good thermal and chemical stability [26] and abundant functionalities [23], are closely linked to their chemical composition and structure. ZIF-67 is a cobalt-based zeolitic imidazolate framework whose structure consists of Co^2+^ ions interconnected with the organic compound 2-methylimidazole (Hmim) and possesses cubic crystal symmetry [27,28]. The method of synthesis and reactant concentrations play an important role in controlling not only the type of nanostructure obtained but also the particle size of ZIF-67 [29,30,31,32,33]. The structure and morphology of ZIF-67 allow a wide range of applications. Due to its high porosity, large specific surface area, and accessible adsorption sites, ZIF-67 has attracted considerable attention as an adsorbent for the removal of various organic pollutants, particularly cationic dyes such as methylene blue [34] and crystal violet [35]. Its applications also include separation of gases [36], gas storage [37], electrocatalysts [38] and supercapacitors [39]. The insulating nature and low electrical conductivity of ZIF-67 represent a significant drawback, limiting its application in systems that require high conductivity [40]. For this reason, there is a rising interest in the development of new composites where ZIF is incorporated into other materials, such as conductive polymers [41].

Conductive polymers represent a class of conjugated synthetic polymers characterized by their electrical conductivity, capacity for dopant counterion exchange along the polymer backbone, the ability to change oxidation state (redox activity) and the capability to transition from a conductive state to an insulating state upon alkali treatment (dedoping) [42]. Their electrical, chemical, optical, and mechanical properties, as well as their structure, can be altered by changing the synthesis conditions or applying various post-synthesis treatments [43,44]. Among conductive polymers, polyaniline (PANI) stands out particularly due to its simple synthesis, good environmental stability, low cost, high conductivity, which can be easily controlled, and possibility to form different nanostructures [45]. Conducting PANI nanotubes and nanorods have previously been synthetized using 5-sulfosalicylic acid as a dopant and ammonium peroxydisulfate as an oxidant [46], while PANI nanofibers were produced in bulk by chemical oxidative polymerization [47]. The limitation regarding the low specific surface area of PANI can be overcome by facile synthesis of composites with various metal–organic frameworks (MOFs), resulting in a material possessing properties derived from both PANI and MOF. It has been reported that composites of PANI with MOF-5 represent new class of materials with high surface area, relatively high conductivity and high microporosity [48].

Beyond the increase in surface area achieved by combining MOFs with conductive polymers, the rational integration of both components can introduce complementary adsorption functionalities within a single hybrid system. In this regard, a synergistic approach for dye removal was applied here through developed ZIF-67/PANI-SSA composites, where the high porosity of ZIF-67 and its ordered structure with N-containing aromatic rings in Hmim ligand are complemented by N-containing functional groups, counter-ions and the aromatic, conjugated structure of PANI-SSA, which may provide new active centers, modify surface charge and promote additional interactions with aromatic dye molecules, such as BF. Thus, this hybrid architecture combines the structural advantages of the MOF with the chemically active functionalities of the polymer. However, the preparation of MOF/conductive polymer composites requires careful consideration of the compatibility between both components and preservation of the MOF structure, particularly when polymer formation involves conditions that may affect MOF stability. Therefore, simple, mild, and solvent-minimized approaches to their preparation are of particular interest.

Among numerous approaches for the synthesis of composites, the mechanochemical approach has received increased attention in recent years. The mechanical force allows grinding and efficient mixing of components to produce a composite. Mechanochemical syntheses of composites have several important advantages: the procedure is simple, practical, relatively short, eco-friendly and more sustainable, as it enables avoiding or diminishing the use of solvents. The mechanochemical approach has been used for syntheses of PANI composites with various compounds [49], such as MOF-5 [48], TiO_2_ [50], montmorillonite [51], and carbon materials [52]. In the case of compounds whose stability in a solution is poor, mechanochemical synthesis can be the method of choice.

In this paper, novel ZIF-67/PANI-SSA composites were synthesized using a mechanochemical approach—liquid-assisted grinding, using a small amount of added solvent, ethanol. This method was chosen due to its simplicity and reliability in terms of preserving the structure of ZIF-67, taking into account that the common procedure of oxidative polymerization of aniline to produce PANI requires an acidic aqueous environment in which ZIF-67, if added, could lose its stability, as indicated by our preliminary experiments. It is known that ZIF-67 has limited chemical stability in aqueous environments, especially under acidic conditions, due to hydrolysis of Co−N coordination bonds, although the method/conditions of its synthesis and various modifications affect and can improve its stability [53]. Generally, ZIF-67 is stable in alkaline aqueous solutions and common organic solvents, like methanol, ethanol and acetone.

Synthesized ZIF-67/PANI-SSA composites were subsequently characterized to evaluate their structure, morphology, composition, thermal stability, textural properties and electrical conductivity, and their properties were compared with those of pure ZIF-67 and PANI-SSA. The effects of ZIF-67 content on the overall characteristics of the composites were systematically investigated. The composites obtained were further examined for their ability to adsorb BF dye from an aqueous medium, aiming to explore their potential application in wastewater treatment. All composites behaved as excellent adsorbents, and the one synthesized with 75 wt.% ZIF-67 exhibited the highest removal efficiency and adsorption capacity, which even exceeded that of pure ZIF-67. This study provides a deeper understanding of how the incorporation of ZIF-67 influences the structural, electrical, thermal and adsorption properties of PANI-based materials, highlighting their promise as multifunctional, cost-effective adsorbents for environmental remediation.

## 2. Results and Discussion

### 2.1. Electrical Conductivity and Cobalt Content

**Figure 1:**
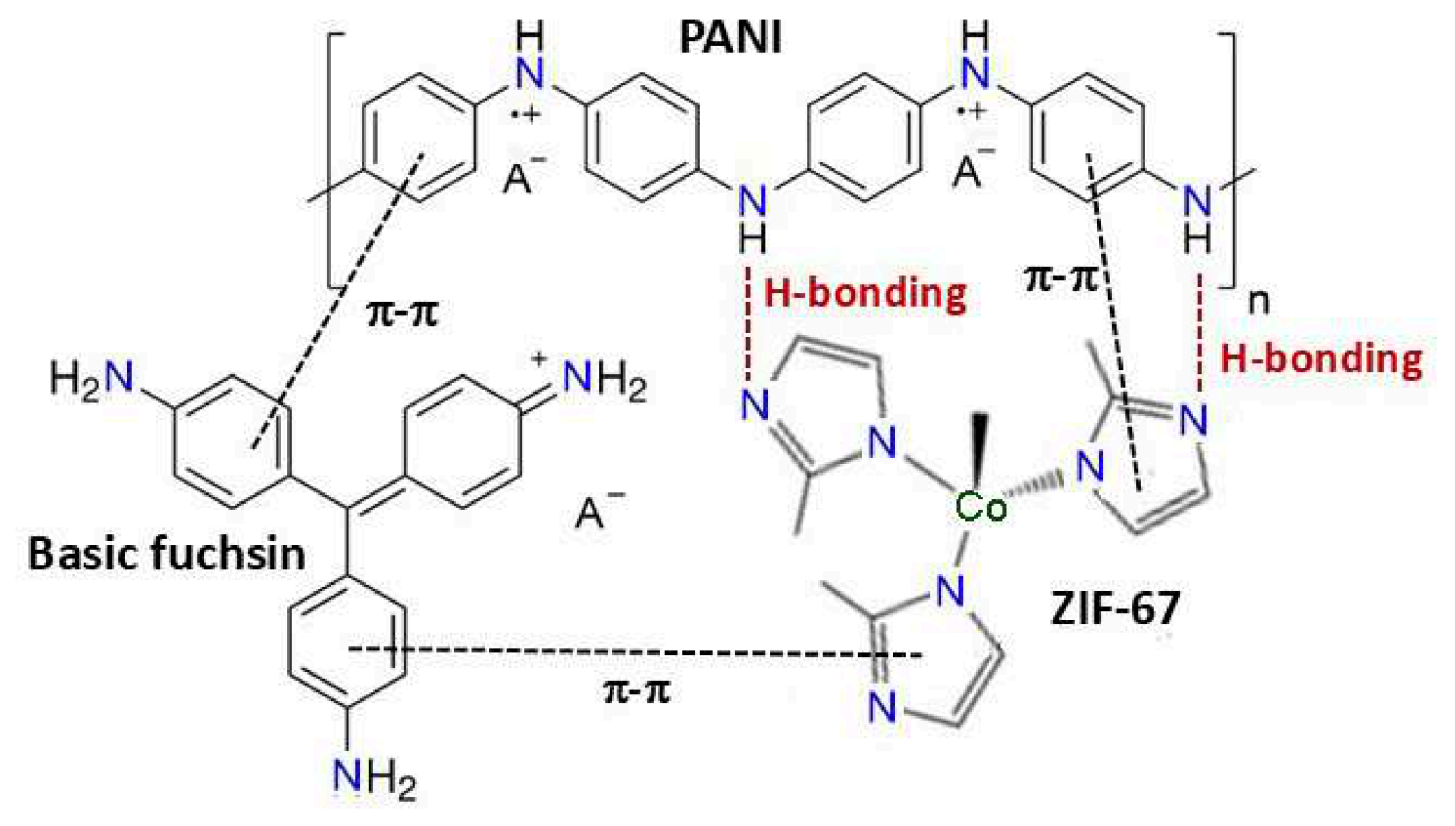
Possible hydrogen bonding and π–π interactions between PANI (in its polaron, conducting form) and ZIF-67 in ZIF-67/PANI-SSA composites, as well as π–π interactions of BF dye with both PANI and ZIF-67 components of composites; A^−^: SSA anions (counter-ions) from PANI-SSA and Cl^−^ anions from BF.

The electrical conductivity and cobalt content of the synthesized PANI-SSA and ZIF-67 precursors and ZIF-67/PANI-SSA composites are summarized in Table 1. ZIF-67 exhibits a very low conductivity of 2.89 × 10^−8^ S cm^−1^, confirming its insulating nature [54]. On the other hand, PANI in its polaronic, emeraldine salt form (Figure 1) is conductive. Comparative analysis of the conductivity values for pure PANI-SSA (3.4 × 10^−2^ S cm^−1^) and the composites (10^−5^–10^−6^ S cm^−1^) shows that the inclusion of ZIF-67 results in a progressive reduction in electrical conductivity. This decrease is attributed to the physical distribution of insulating ZIF-67 particles on PANI nanostructures, which disrupts π-conjugation pathways and hinders charge carrier mobility. Among the composites, the sample with the highest PANI content exhibited the greatest electrical conductivity, 1.3 × 10^−5^ S cm^−1^, while other composites have conductivity of order 10^−6^ S cm^−1^. The enhanced electrical conductivity of the composites compared to pure ZIF-67, whose inherently low conductivity results from the insulating organic imidazolate framework, is due to the conductive network established by PANI-SSA chains [55].

The cobalt in the samples originates from ZIF-67 (Figure 1), and its content was determined by FAAS (Table 1). It amounted to 23.2 wt.% for pure ZIF-67 and increased for the composites from 4.7 wt.% to 22.0 wt.%, with increasing ZIF-67 content from 20 wt.% to 75 wt.%, respectively, indicating successful integration of ZIF-67 and PANI into composites.

### 2.2. Morphology—SEM

**Figure 2:**
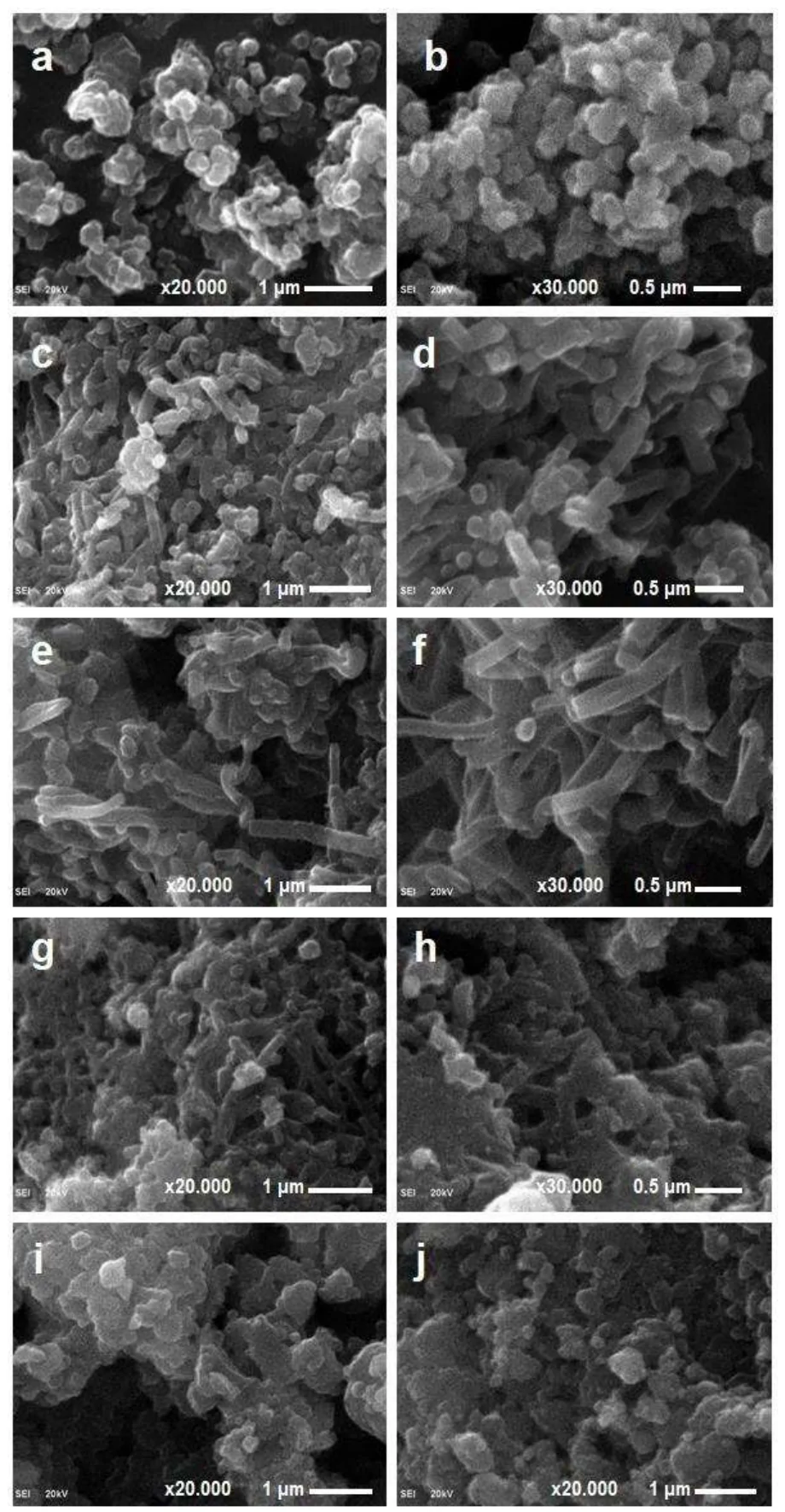
SEM images of (**a**,**b**) ZIF-67, (**c**,**d**) PANI-SSA, (**e**,**f**) 20%ZIF-67/PANI-SSA, (**g**,**h**) 25%ZIF-67/PANI-SSA, (**i**) 50%ZIF-67/PANI-SSA, and (**j**) 75%ZIF-67/PANI-SSA.

SEM images of ZIF-67, PANI-SSA and ZIF-67/PANI-SSA composites are shown in Figure 2.

Pure ZIF-67 contains mostly irregular cuboidal submicron particles (edge length c.a. 0.1–0.5 μm), with rounded corners and edges (Figure 2a,b), as it has been observed in our previous study [56]. The morphology of pure PANI-SSA consists mainly of one-dimensional (1-D) particles, nano/submicron rods or tubes, with a diameter of c.a. 100–200 nm and a length of c.a 0.4–2 μm (Figure 2c,d), similarly to the morphology of PANI-SSA in ref. [46]. SEM images of ZIF-67/PANI-SSA composites (Figure 2e–j) show significant changes in their morphology with increasing ZIF-67 content. In the composite synthesized with the lowest amount of ZIF-67 (20 wt.%), 1-D PANI particles are prevailing, whereas cuboidal ZIF-67 particles can also be seen (Figure 2e,f). Spongy morphology composed of ZIF-67 and 1-D PANI particles linked together and forming a network, with the presence of larger, flattened regions of agglomerated particles, is characteristic of the composite with 25 wt.% of ZIF-67 (Figure 2g,h). For the composites with higher ZIF-67 content, 50 wt.% (Figure 2i) and 75 wt.% (Figure 2j), granular morphology is dominating, while PANI-SSA 1-D nanostructures are barely observed as being covered by prevalent ZIF-67 particles. Flattened larger structures/aggregates with higher packing density, formed due to high-shear forces during mechanochemical synthesis, are also present in these two composites. SEM images indicate effective mixing of the two components via the physical blending process.

### 2.3. Thermogravimetric Analysis (TGA)

**Figure 3:**
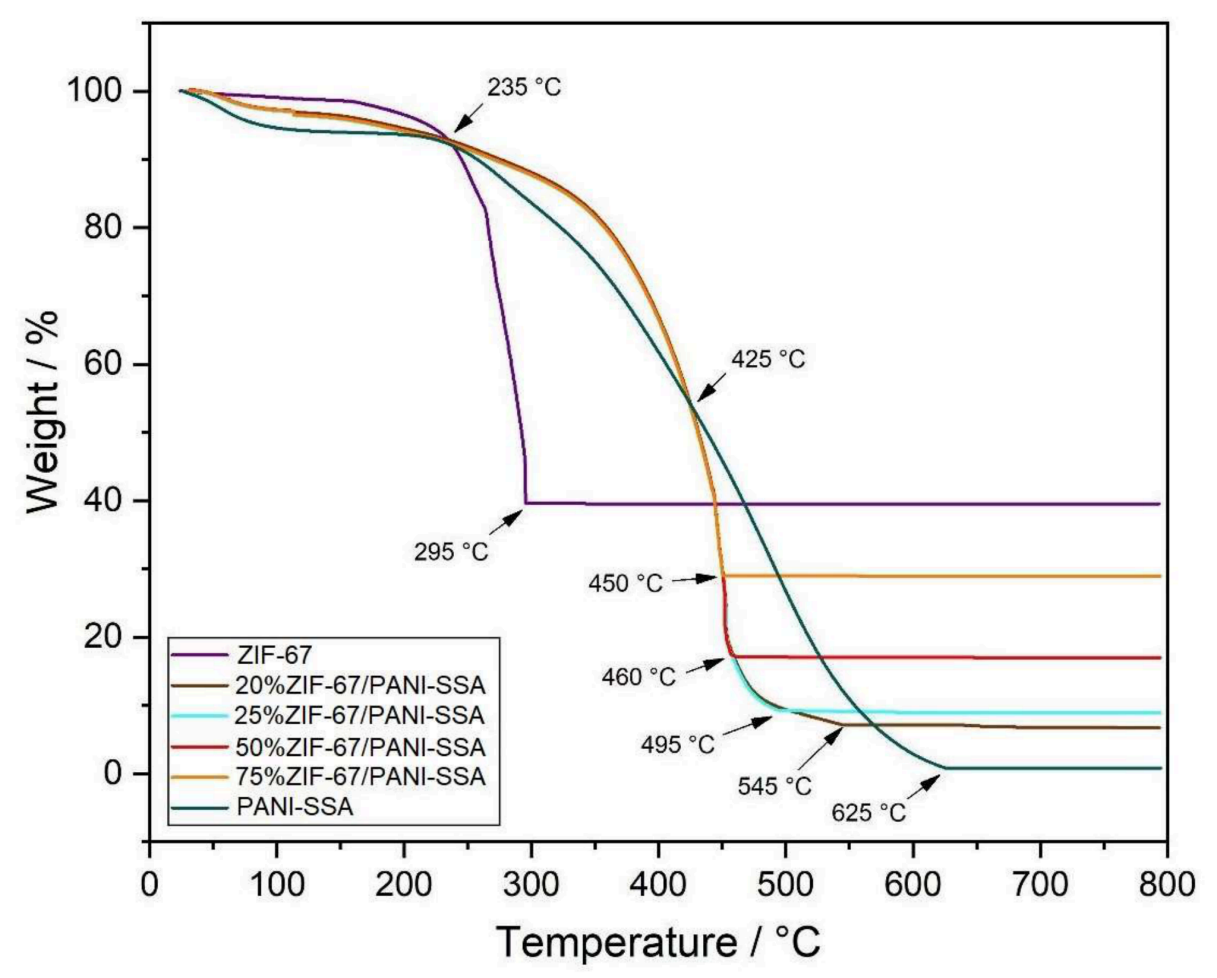
TGA curves for ZIF-67, PANI-SSA and ZIF-67/PANI-SSA composites, recorded in a synthetic air flow.

Thermal degradation of ZIF-67, PANI-SSA and ZIF-67/PANI-SSA composites in a synthetic air atmosphere was investigated using TGA (Figure 3). Table 2 presents data on the weight loss and weight residue for these materials determined from TGA curves. The initial weight loss observed between 25 °C and approximately 200 °C for all samples is attributed to the desorption of physically adsorbed, residual water [57,58,59]. This step is relatively small and confirms that no significant structural decomposition occurs at low temperatures. The smallest amount of water is adsorbed by pure ZIF-67, somewhat higher by ZIF-67/PANI-SSA composites (which increases with increasing PANI-SSA content in them) and the highest by pure PANI-SSA (Table 2), indicating that ZIF-67 is the least hygroscopic among the samples. Pure ZIF-67 shows rapid weight loss in the temperature range from 235 to 295 °C, which is associated with the breakdown of coordination bonds between cobalt ions and imidazolate linkers, leading to structural collapse of the framework, and the decomposition of the organic linker, finally resulting in thermally stable Co_3_O_4_ [60,61,62]. At temperatures above 295 °C, almost no further weight loss is observed, and the weight residue at 800 °C is about 39% (Table 2), which is consistent with literature data on the thermal transformation of ZIF-67 into Co_3_O_4_ [60,61]. On the other hand, pure PANI-SSA displays much slower decomposition starting at ~235 °C and ending at 625 °C, almost without residue. This main weight loss in PANI-SSA is primarily due to the thermal degradation and decomposition of the PANI backbone [59,63]. TGA curves for ZIF-67/PANI-SSA composites have the shape somewhere between that for pure ZIF-67 and PANI-SSA. Thermograms of the composites almost coincide with each other in the temperature range 200−450 °C. Interestingly, in the temperature range between 235 and 425 °C, all composites show better thermal stability (slower weight loss) than both individual ZIF-67 and PANI-SSA, indicating synergistic action of the two composite components. It can be supposed that the framework and crystal structure of ZIF-67 make decomposition of PANI components in the composites more difficult, whereas long PANI chains interact with small, nearby imidazolate linkers and protect them from thermal decomposition. Both aniline units in PANI-SSA and imidazolate rings in ZIF-67 are aromatic and contain nitrogen, so they can interact via hydrogen bonds, such as N−H…N or =N−H^+^ …N, and/or π–π interactions (Figure 1). Also, all composites show better thermal stability than pure ZIF-67 manifested in higher temperatures of complete decomposition/transformation to Co_3_O_4_ (above which there is almost no further weight loss), which amounted to c.a. 450, 460, 500 and 550 °C for composites synthesized with 75, 50, 25 and 20 wt.% of ZIF-67, respectively (as marked by arrows in Figure 3), while for ZIF-67 the temperature of complete degradation is 295 °C. This trend means that the thermal stability of the composites increases with increasing PANI content in them. The weight of final residue at 800 °C (associated mainly with formed Co_3_O_4_) for composites increases with increasing ZIF-67 content, as expected (Table 2).

### 2.4. FTIR Spectroscopy

**Figure 4:**
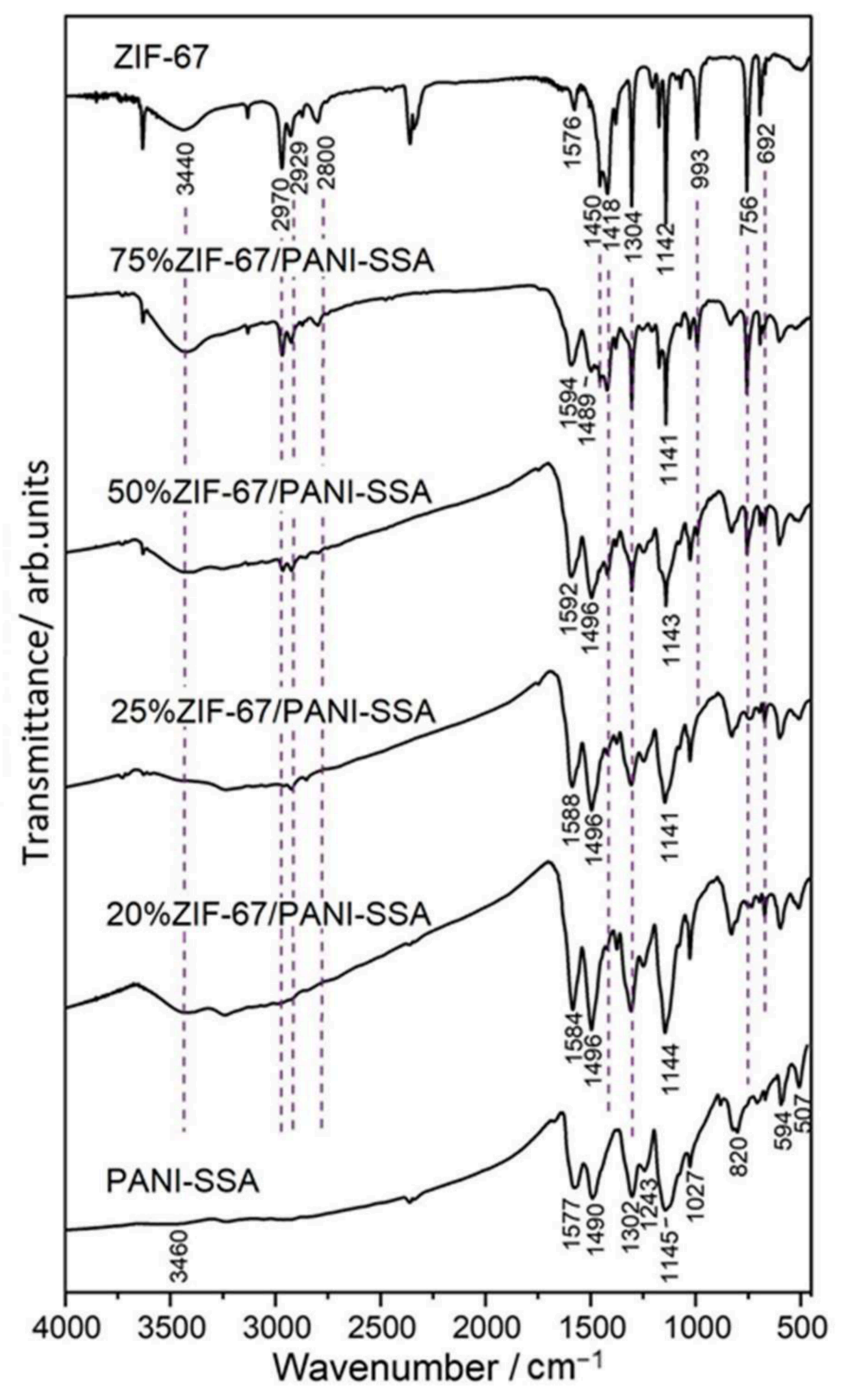
FTIR spectra of ZIF-67, PANI-SSA and ZIF-67/PANI-SSA composites.

The FTIR spectra of ZIF-67/PANI-SSA composites as well as pure PANI-SSA and ZIF-67 are shown in Figure 4. All prepared composites display characteristic bands of a conducting, emeraldine salt form of PANI (Figure 1). The characteristic PANI bands at ~1580 cm^−1^, assigned to the C=N and C=C stretching vibrations of the quinonoid (Q) ring, and at ~1490 cm^−1^, corresponding to the aromatic C–C stretching vibration of the benzenoid (B) ring, are clearly observed in the FTIR spectra of all composites [46,64]. Compared to pure PANI-SSA (1577 and 1490 cm^−1^), these bands are slightly shifted toward higher wavenumbers in the spectra of composites. This blueshift increases for the ~1580 cm^−1^ band with increasing ZIF-67 content in composites, 1584, 1588, 1592, and 1594 cm^−1^ for 20, 25, 50, and 75 wt.% of ZIF-67, respectively, indicating strong interaction between the two composite components and partial dedoping of PANI chains [65]. The band positioned at ~1300 cm^−1^ is associated with the C–N stretching ν(C–N_sec_) of secondary aromatic amine [64], while the band at ~1240 cm^−1^ is attributed to the C–N^+•^ stretching vibration ν(C–N^+•^)_polaron_ in the polaron structure (Figure 1) and is indicative for PANI electrical conductivity [65]. The band appearing at ~1140 cm^−1^ corresponds to the B–NH^+^=Q and B–NH^+•^–B stretching modes and in-plane C–H bending vibration, δ(C–H), and its intensity was also correlated with the degree of protonation, electron delocalization and conductivity of PANI [46,65,66,67]. With increasing ZIF-67 content, the relative intensity of the band at ~1240 cm^−1^ gradually decreases and is lowest in the spectrum of composite containing 75 wt.% ZIF-67 where this band is very weak. This feature indicates partial suppression of the polaronic C–N^+•^ stretching vibration, likely resulting from interactions between the PANI-SSA chains and the ZIF-67 framework. A large amount of ZIF-67 component reduces the doping level and charge delocalization in chains, hindering interchain conducting pathways, which is consistent with measured electrical conductivity (Table 1). Pronounced alterations are also observed in the intensity and shape of the bands around ~1300 and ~1140 cm^−1^. These bands progressively narrow as the ZIF-67 content increases, which may be attributed to changes in the local electronic environment of the PANI backbone, as well as overlapping contributions from the ν(C–N) and vibration of the Hmim ligand in ZIF-67, which occurs as very strong and sharp at 1304 cm^−1^ in the spectrum of pure ZIF-67. The band at 820 cm^−1^ represents an aromatic out-of-plane C–H bending vibration in the linear PANI backbone [68]. The band at ~1025 cm^−1^ is attributed to the SO_3_ symmetric stretching of SSA anions, and the band at 595 cm^−1^ is assigned to the out-of-plane bending of the SSA ring [46,64,69]. In the spectra of all composites, the bands due to hydrogen-bonded N–H stretching vibrations (“H-peaks”) are observed in the 3300–2800 cm^−1^ region [70,71]. The composites show a broad band centered at c.a. 3460 cm^−1^ assigned to the N−H stretching vibrations of the secondary amine in the PANI backbone [71] and imidazole ligands of the ZIF-67, which is strongest in the spectrum of 75%ZIF-67/PANI-SSA. The band at 3250 cm^−1^ is attributed to the stretching vibrations of hydrogen-bonded N−H and/or =N−H^+^ bonds in PANI [71], and its relative intensity decreases with increasing ZIF-67 content in composites.

The FTIR spectra reveal that most of the characteristic vibrational bands of ZIF-67, which were well defined in the pure ZIF-67 spectrum, remain visible for the composites but display notably lower intensities. The bands positioned at ~2970 and ~2929 cm^−1^ are associated to the asymmetric and symmetric C–H stretching vibrations of the methyl group on the imidazole ring, respectively [70]. The band at ~1578 cm^−1^ can be attributed to the stretching vibrations of C=N in the Hmim ligand [72,73], while a band at 1420 cm^−1^ is assigned to the C=C vibration in the imidazole ring [72,74]. Bending vibration from C–N is identified at wavenumber 990 cm^−1^ [75]. Absorption bands at 754 cm^−1^ and 692 cm^−1^ can be assigned to the C–H out-of-plane bending and the ring out-of-plane bending vibration of the Hmim ligand, respectively [76]. The presence of these bands in the composite spectra, albeit with reduced intensity compared to pure ZIF-67, indicates the successful incorporation of ZIF-67 between PANI-SSA chains via the mechanochemical method, maintaining its structural integrity.

### 2.5. Raman Spectroscopy

**Figure 5:**
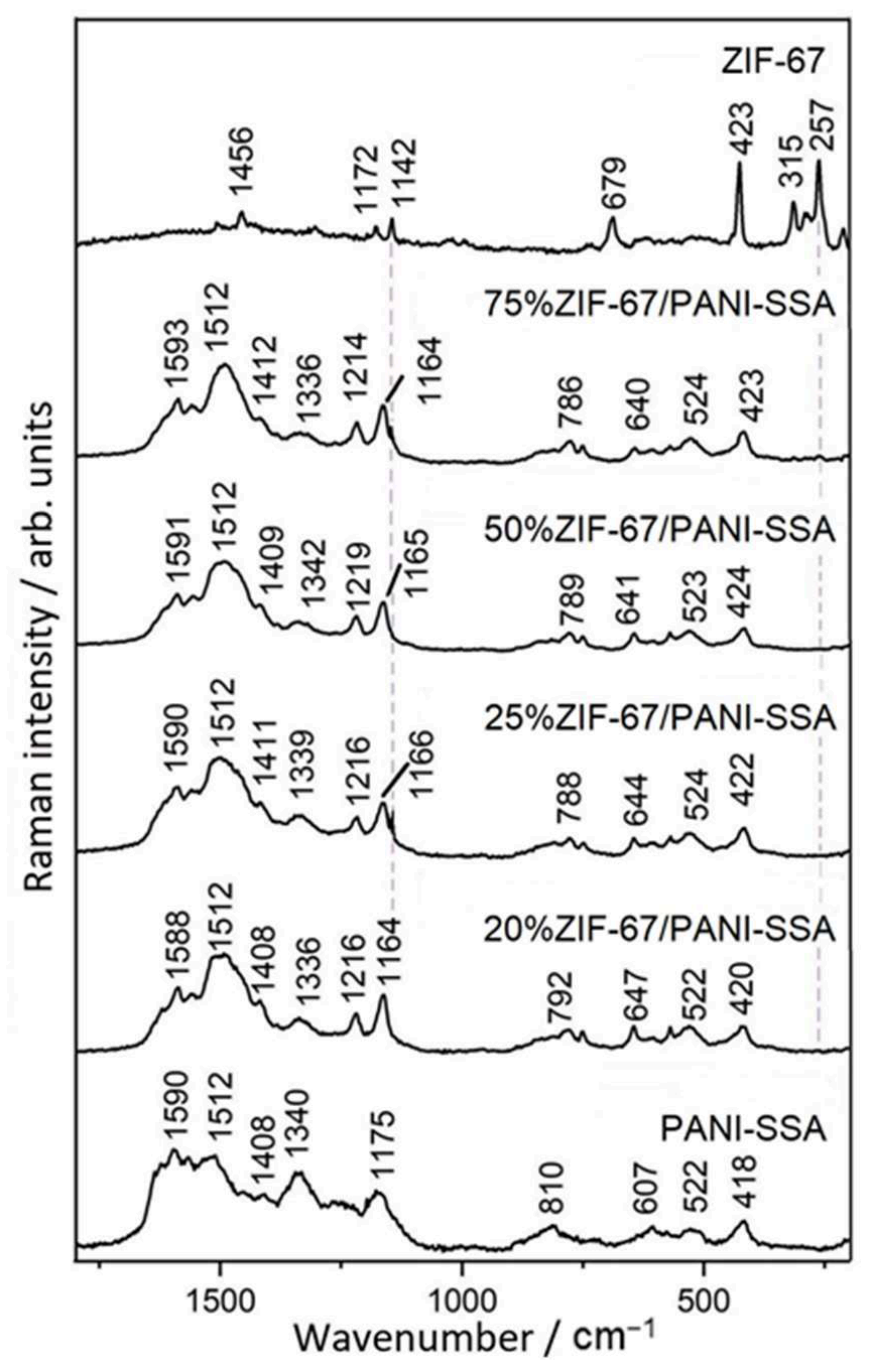
Raman spectra of ZIF-67, PANI-SSA, and ZIF-67/PANI-SSA composites.

Raman spectra of PANI-SSA, ZIF-67 and all ZIF-67/PANI-SSA composites are shown in Figure 5. The Raman spectrum of ZIF-67 is characterized by a small number of bands, primarily associated with vibrational modes of the imidazole ring and the –CH_3_ group. The bands at approximately 1172 and 1142 cm^−1^ are attributed to C–N stretching vibrations, while the band at around 1456 cm^−1^ corresponds to C–H vibrations [77,78]. The bands observed near 257 and 679 cm^−1^ arise from imidazole ring vibrations, whereas those at approximately 286 and 423 cm^−1^ are assigned to Co–N vibrational modes [78,79]. ZIF-67 exhibits a limited number of Raman-active vibrational modes, especially within the 1700–800 cm^−1^ range. Therefore, the bands detected in this region for ZIF-67/PANI-SSA composites are primarily attributable to PANI-SSA, facilitating a more accurate evaluation of structural and electronic changes in the polymer induced by composite formation. In the Raman spectrum of PANI-SSA, a shoulder present at ~1627 cm^−1^ is assigned to the C–C stretching vibration of the benzenoid (B) ring, ν(C–C)_B_ [80]. The strong band at 1590 cm^−1^ is attributed to the C=C stretching vibration in the quinonoid ring (Q), ν(C=C)_Q_, and C~C stretching vibrations of the semi-quinonoid (SQ) rings, ν(C~C)_SQ_, where ~ denotes the bond intermediate between the single and double bonds [57,81], while two bands at ~1512 cm^−1^ and ~1340 cm^−1^ correspond to the N–H deformation vibrations of protonated amine δ(N–H) and C~N^+•^ stretching vibration of delocalized polaronic structures, respectively [82]. The strong band is at ~1340 cm^−1^ [83]. The band observed at ~1408 cm^−1^ is assigned to phenazine-type units in PANI units [84]. The band at ~1175 cm^−1^ is due to the in-plane C–H deformation of SQ rings, δ(C–H)_SQ_. The weak band positioned at ~1266 cm^−1^ is associated with the benzenoid ring deformation vibrations [80]. The band at ~810 cm^−1^ is due to the C–H deformation of the Q ring, δ(C–H)_Q_, while the band at ~607 cm^−1^ may be attributed to the in-plane deformation of the benzenoid ring (B) and C–S stretching vibration [82].

In the Raman spectra of the ZIF-67/PANI-SSA composites, several notable changes are observed compared to the spectrum of pure PANI-SSA. The intensity ratio of the bands at c.a. 1510 and 1590 cm^−1^ is inverted in the spectra of composites: the 1510 cm^−1^ band becomes more intense than the 1590 cm^−1^ band, and the broadening of the band at 1510 cm^−1^ is observed, due to the appearance of a new component/shoulder at about 1490 cm^−1^, which corresponds to the C=N stretching vibration in quinonoid units [84]. These features indicate a shift in electron density along the polymer chain, reduced π-electron delocalization, and decreased content of semiquinonoid units [85]. This reflects a partial dedoping of PANI [45], which can occur by the interactions (e.g., hydrogen bonding −NH^+•^…N) with basic imidazolate ligands in ZIF-67, acting as a deprotonating agent via their uncoordinated nitrogen atoms. The dedoping process is likely facilitated by Co^2+^ ions from ZIF-67 which can coordinatively interact with nitrogen atoms in PANI chains [86]. The intensity of the band at ~1336 cm^−1^, associated with C–N^+^ stretching vibrations of polaronic structures, decreases, which is an additional indication of fewer polaronic structures and the partial dedoping of PANI. Additionally, the bands at 1216 and 1171 cm^−1^, corresponding to C–H bending and C–N stretching vibrations, become narrower, suggesting a more uniform electronic environment along the chain [87]. Compared to pure PANI-SSA, bands at 1175 cm^−1^ and 810 cm^−1^ are slightly shifted toward lower wavenumbers in the composites. The intensity of the band at ~810 cm^−1^, assigned to the C–H deformation of quinonoid rings, decreases, while the intensity of the band at ~790 cm^−1^, associated with C–H deformation of benzenoid rings, increases, indicating partial conversion of quinonoid units into benzenoid units in the polymer backbone in composites [87].

### 2.6. Textural Properties—N_2_ Adsorption Analysis

**Figure 6:**
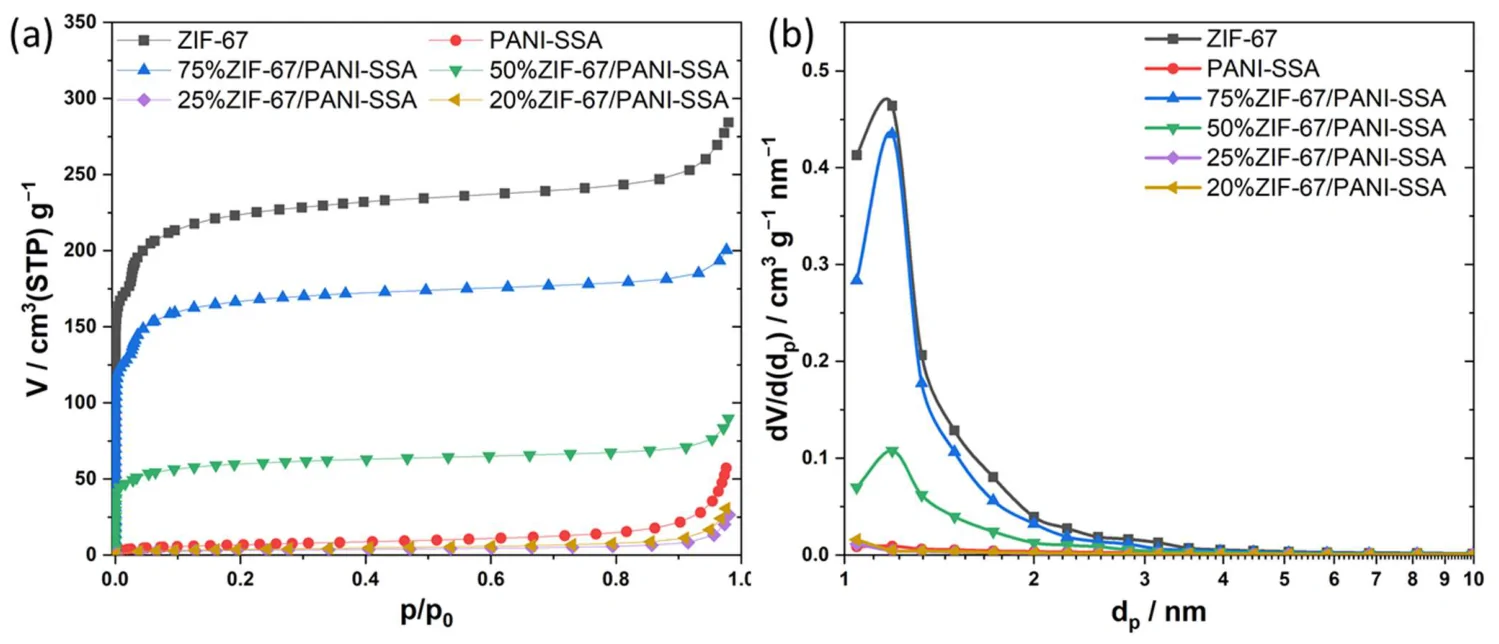
(**a**) N_2_ adsorption isotherms and (**b**) BJH pore size distributions for pure PANI-SSA, pure ZIF-67, and ZIF-67/PANI-SSA composites. Note that, because the BJH distributions were presented for pore diameters above 1 nm, features between 1 and 2 nm were interpreted only qualitatively.

The N_2_ adsorption–desorption isotherms reveal a pronounced composition-dependent transition from ZIF-67-dominated microporosity to the predominantly non-microporous texture of PANI-SSA (Figure 6). Table 3 summarizes textural properties of the studied materials. The ZIF-67 framework exhibited a high Brunauer–Emmett–Teller specific surface area (S_BET_) of 841 m^2^ g^−1^ and a total pore volume (V_tot_) of 0.440 cm^3^ g^−1^. Incorporation of 25 wt.% PANI-SSA preserved the porous structure remarkably well. The 75%ZIF-67/PANI-SSA composite reached S_BET_ of 651 m^2^ g^−1^, close to the value of 637 m^2^ g^−1^ expected from a simple mass-weighted combination of the two components. Its micropore surface area obtained by the Mikhail–Brunauer–Bodor method, i.e., the MP method (see Section 3), was 713 m^2^ g^−1^, and the volume was 0.259 cm^3^ g^−1^, and these were likewise comparable to the ZIF-67 fraction expected in the composite, demonstrating that most of the accessible ZIF-67 microporosity was retained. A substantially non-linear decrease was observed at higher PANI-SSA contents. The S_BET_ of the 50%ZIF-67/PANI-SSA composite was only 228 m^2^ g^−1^, approximately 53% of the mass-weighted value, while its MP volume decreased to 0.080 cm^3^ g^−1^. No measurable microporosity was detected for the 25% and 20% ZIF-67 composites, whose S_BET_ values of 11.0 and 13.7 m^2^ g^−1^ were even lower than that of PANI-SSA itself (24.9 m^2^ g^−1^). These results indicate that the loss of accessible porosity cannot be explained solely by dilution of ZIF-67 and is consistent with progressive pore blocking by PANI-SSA and/or mechanochemically induced disruption of the ZIF-67 framework.

The Barrett–Joyner–Halenda (BJH) analysis further illustrates this textural transition. ZIF-67 and the 75%ZIF-67/PANI-SSA composite showed apparent average pore diameters d_av_ of 1.68 and 1.58 nm, respectively, whereas the 50% composite shifted toward the mesopore boundary d_av_. PANI-SSA and the composites containing 25% and 20% ZIF-67 displayed substantially larger average diameters of 8.52, 5.74, and 6.20 nm, respectively, which are attributed mainly to interparticle voids rather than intrinsic framework pores.

### 2.7. Zeta Potential

The mean value of the zeta potential determined for 75%ZIF-67/PANI-SSA composite (the best adsorbent) from three measurements was −14.1 ± 0.8 mV, indicating a weakly negative overall electrokinetic potential under the applied measurement conditions. The negative surface charge is expected to promote the adsorption of BF, cationic dye, through electrostatic attraction between the negatively charged adsorbent surface and positively charged dye molecules. The measured zeta potential reflects an average electrokinetic response of the composite surface and does not imply a uniformly charged surface. ZIF-67-rich and PANI-SSA-rich regions may retain different local surface charge characteristics. Consequently, the measured value of −14.1 mV should be interpreted as the resulting response of a chemically and electrostatically heterogeneous composite surface.

According to previous reports [88], pristine ZIF-67 exhibits a near-neutral or slightly positive zeta potential at the pH of the sample dispersion (pH 6.5), becomes more positively charged with increasing pH, and shifts to negative values under strongly alkaline conditions. Therefore, the negative zeta potential observed for the ZIF-67/PANI-SSA composite cannot be attributed to the intrinsic surface charge of pristine ZIF-67 under comparable aqueous conditions. Instead, it suggests that incorporation into the PANI-SSA matrix modifies the surface and interfacial electrostatic environment of the composite. Partial dedoping of PANI-SSA, indicated by FTIR and Raman analyses, reduces the density of positively charged protonated nitrogen sites along the polymer backbone, while negatively charged SSA counter-ions (Figure 1) may also contribute to the surface charge. Therefore, the interactions between ZIF-67 and PANI-SSA may shift the overall electrokinetic potential of the composite towards negative values.

### 2.8. Dye Adsorption Study

#### 2.8.1. Effect of Contact Time on the Adsorption Process for BF Dye

**Figure 7:**
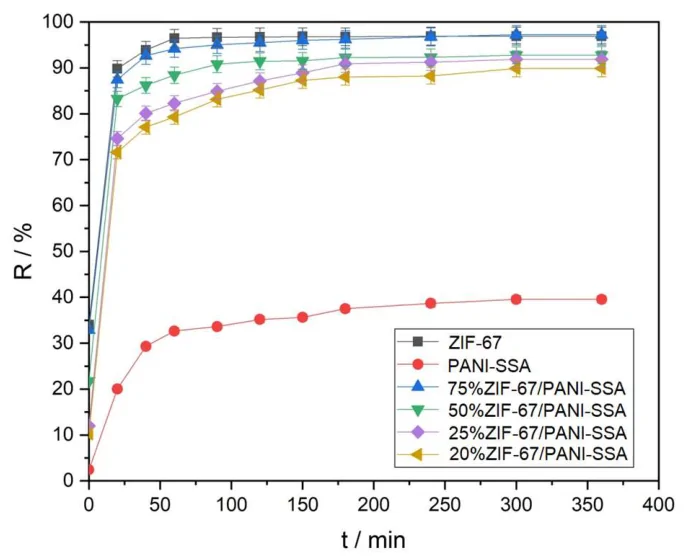
Effect of contact time (t) on the adsorption of BF dye on different samples. The concentration of adsorbent in a suspension was 1.0 g L^−1^. The initial concentration of BF was 100 mg L^−1^, and the temperature was 298 K.

The study of the contact time effect on the adsorption of BF dye was carried out for all samples (Figure 7). ZIF-67 provides a high surface area and a microporous framework, offering abundant sites for dye molecules to be trapped [56]. PANI-SSA contributes nitrogen-containing functional groups and π-conjugated chains, which can interact with the cationic dye through electrostatic attractions and π–π interactions [89]. For malachite green, the dye with a similar structure to BF, π–π interactions were proposed as the most important for its adsorption on ZIF-67 [90]. The quick absorption of the dye can be noticed in the first 60 min of the adsorption process, which is due to the presence of a high number of empty adsorbent sites. The adsorption rate becomes slower after 2 h, and equilibrium was reached after 180 min of shaking the solution of BF dye and adsorbents. A 360 min contact time was used as a reference to ensure that equilibrium was reached and the removal efficiency R approached the constant values, 96.9, 39.5, 97.2, 92.7, 91.9 and 89.9% for ZIF-67, PANI-SSA, 75%ZIF-67/PANI-SSA, 50%ZIF-67/PANI-SSA, 25%ZIF-67/PANI-SSA and 20%ZIF-67/PANI-SSA, respectably (Figure 6). Notably, all composites are much better adsorbents for BF than pure PANI-SSA and have comparable removal efficiency to ZIF-67. A synergistic adsorption effect is evident for all composites, as their maximum capacities consistently exceed the values predicted from the mass-weighted capacities of the individual PANI-SSA and ZIF-67 components. The effect is most pronounced for the composites containing 20 and 25% ZIF-67, which show the largest positive deviations despite having the lowest specific surface areas (Table 3) and distinct 1-D nanostructure morphology (Figure 2), resulting in the highest maximum adsorption capacities per unit surface area and indicating the greatest density of accessible adsorption sites. By contrast, the superior overall capacity of 75%ZIF-67/PANI-SSA primarily reflects its much larger surface area, combined with less pronounced yet still significant synergistic interactions between PANI-SSA and ZIF-67.

#### 2.8.2. Adsorption Kinetics

**Figure 8:**
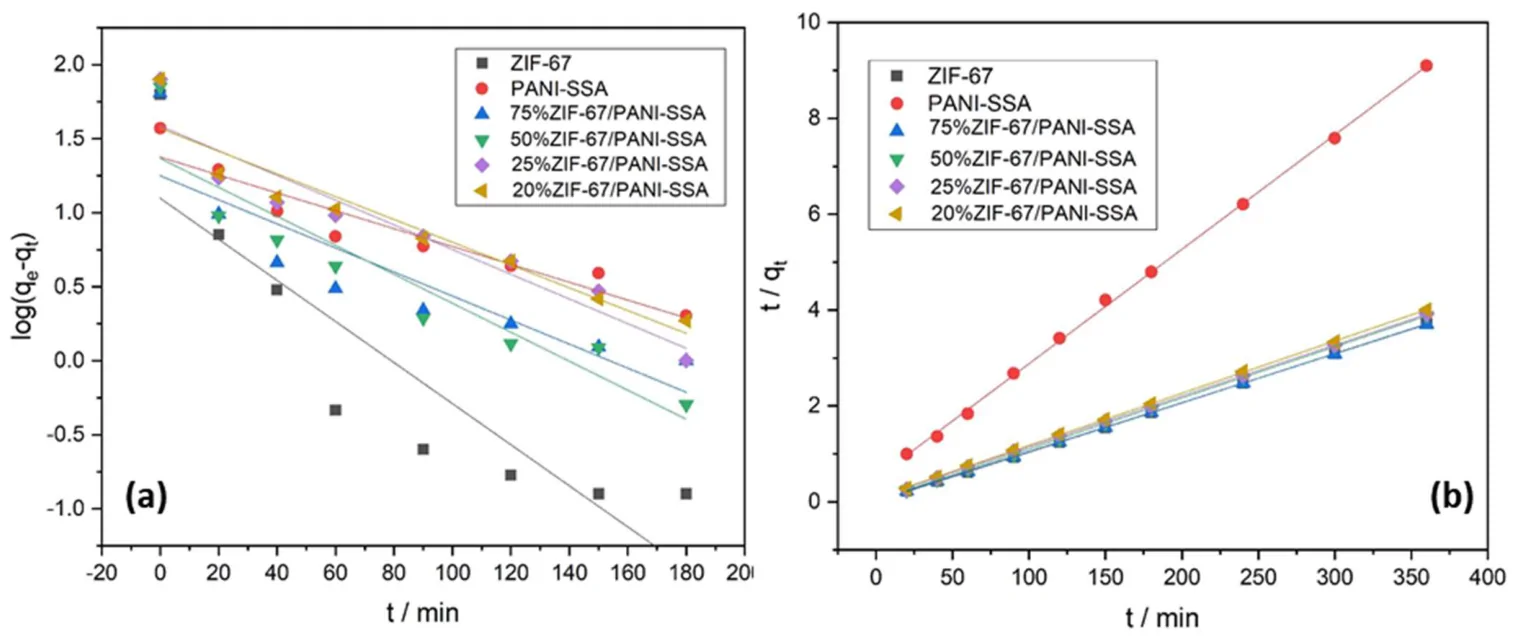
(**a**) Pseudo-first-order kinetic plots and (**b**) pseudo-second-order kinetic plots for adsorption of BF on ZIF-67, PANI-SSA and ZIF-67/PANI-SSA composites.

Figure 8a,b shows the pseudo-first-order and the pseudo-second-order kinetic plots, respectively, for BF adsorption on the synthesized ZIF-67, PANI-SSA, and ZIF-67/PANI-SSA composites. Comparison was made between experimental and calculated adsorption. Ultra-high adsorption for all the samples indicates a close agreement for the pseudo-second-order kinetic model. Furthermore, the adsorption process is well described by this model (Figure 8b), as supported by the correlation coefficient values reported in Table 4.

#### 2.8.3. Adsorption Isotherms

**Figure 9:**
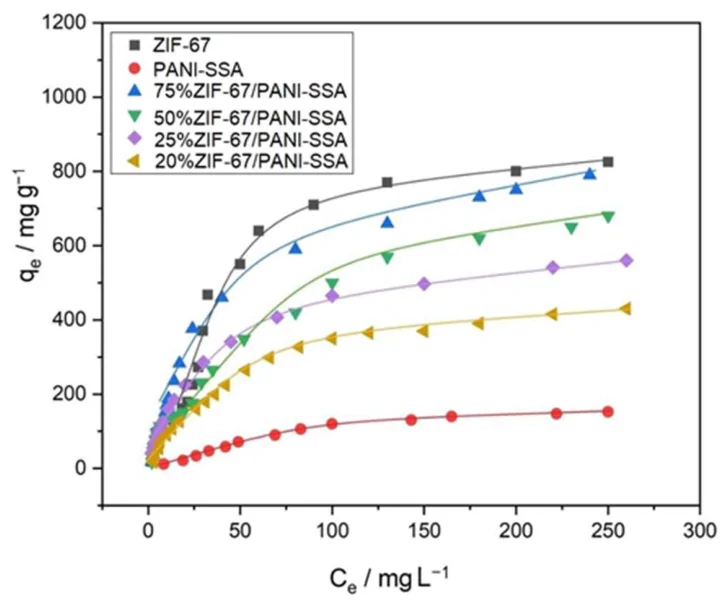
Adsorption isotherms fitted using the Langmuir–Freundlich isotherm model for the adsorption of BF on ZIF-67, PANI-SSA and ZIF-67/PANI-SSA composites.

Among the tested models, the Langmuir–Freundlich isotherm (Figure 9) provided the best fit for all investigated materials, as confirmed by the highest correlation coefficients (R^2^ > 0.99, Table 5). This model combines the features of both Langmuir and Freundlich equations and suggests that adsorption occurs on heterogeneous surfaces with energy distribution that tends toward monolayer formation at higher concentrations. The obtained sorption parameters (Table 4) indicate that all materials exhibited favorable adsorption (1/*n* < 1). The 75%ZIF-67/PANI-SSA composite exhibited the highest maximum adsorption capacity among all investigated materials (q_max_ = 910 mg g^−1^), exceeding that of pure ZIF-67 (q_max_ = 900 mg g^−1^) (Table 4). BF is a cationic dye, so its adsorption is favored on negatively charged or electron-rich surfaces. Enhanced adsorption of BF on the ZIF-67/PANI-SSA composite can be rationalized as a direct effect of the partial deprotonation of PANI chains, which increases the number of favorable binding sites for the cationic dye. Raman spectroscopy indicates partial deprotonation (dedoping) of PANI in ZIF-67/PANI composites, meaning that some of the positively charged –NH^•+^ sites in doped PANI are neutralized (transformed to =N–), increasing the density of electron-rich nitrogen and benzenoid sites along the polymer chain. This generates more electron-rich regions, which can interact electrostatically with the positively charged BF molecules, besides hydrogen bonding and π–π interactions (Figure 1). The highest q_max_ and K values obtained for 75%ZIF-67/PANI-SSA composite confirm its superior affinity toward BF dye, reflecting an optimal balance between accessible porous sites and surface functionality, while composites with lower ZIF-67 content showed notably reduced adsorption capacity due to the smaller absolute number of active sites, because of the low surface areas (Table 3). Pure PANI-SSA exhibited the lowest adsorption capacity, mainly due to its limited surface area and fewer available adsorption sites, while pure ZIF-67, though highly porous, showed slightly lower adsorption capacity compared to the composite with 75 wt.% ZIF-67. Therefore, this composite can be considered the most effective adsorbent, combining the advantages of both components and providing a high degree of surface heterogeneity, strong adsorbate–adsorbent interactions, and excellent dye uptake efficiency.

#### 2.8.4. Analysis of Suspension pH Changes During BF Adsorption

To gain further insight into the adsorption behavior of the investigated materials, the changes in suspension pH before and after BF adsorption were analyzed and correlated with the adsorption performance of PANI-SSA, ZIF-67, and ZIF-67/PANI-SSA composites. PANI-SSA produces a strongly acidic suspension of pH 1.3, which increases to 1.7 after contact with dye and then decreases to 1.4 at equilibrium. Under these highly acidic conditions, the nitrogen-containing groups of PANI are extensively protonated, giving the surface a positive charge. This likely causes electrostatic repulsion with the cationic dye molecules and explains the comparatively low adsorption capacity of PANI-SSA (39.5 mg g^−1^). In contrast, ZIF-67 maintains a mildly alkaline pH throughout the experiment (7.7–7.8) and exhibits a substantially higher adsorption capacity of 96.9 mg g^−1^. At this pH, the ZIF-67 surface is expected to be less positively charged, or partially deprotonated, which reduces electrostatic repulsion and promotes interactions with BF dye. In addition to electrostatic effects, its porous structure and accessible adsorption sites probably enable hydrogen bonding and π–π interactions with the aromatic dye. The composite materials show intermediate suspension pH values that increase progressively with ZIF-67 content, from 5.3 for 20% ZIF-67/PANI-SSA to 6.6 for 75% ZIF-67/PANI-SSA. All composites exhibit high adsorption capacities of 89.9–97.2 mg g^−1^, demonstrating that adsorption capacity rises as the acidity of the suspension lowers. The equilibrium pH changes are relatively small for the composites, ranging from −0.02 to +0.12 pH units. This indicates that the composite surfaces are comparatively stable and buffered during adsorption. The small pH increase observed for composites containing 50 wt.% and 75 wt.% ZIF-67 may reflect proton uptake or ion-exchange processes.

#### 2.8.5. Comparative EDX Analysis Before and After BF Adsorption

The elemental compositions of pristine and BF-loaded ZIF-67, 25%ZIF-67/PANI-SSA, and 75%ZIF-67/PANI-SSA determined by EDX mapping are summarized in Table 6. The atomic percentage of Co determined by EDX for pristine ZIF-67 (8.3 at.%) is in good agreement with the theoretical value of 7.7 at.% calculated from the chemical formula of ZIF-67 considering only the elements detectable by EDX (C, N and Co), since hydrogen cannot be detected by this technique. Following BF adsorption, the Co content decreases to 3.4 at.%, consistent with the coverage of the ZIF-67 framework with dye molecules. Given the EDX interaction volume of approximately 1–2 μm in ZIF-67, this reduction in Co content reflects changes throughout the near-surface bulk rather than only the outermost surface. At the same time, the nitrogen content increases from 24.7 to 34.0 at.%, providing further evidence of successful BF adsorption, as the dye is rich in nitrogen-containing functional groups. The successful incorporation of PANI-SSA into the composites is reflected by the increase in carbon content accompanied by a decrease in the relative proportions of nitrogen, oxygen, and cobalt within the EDX sampling volume. As expected, the extent of the decrease in Co depends on the ZIF-67 loading. For composites containing 75 wt.% ZIF-67, the Co content decreases only slightly (8.3 to 7.9 at.%), whereas for composites containing 25 wt.% ZIF-67, a much more pronounced reduction is observed (8.3 to 1.3 at.%). Following BF adsorption, all composites exhibit the same general trend: an increase in carbon and nitrogen contents, corresponding to the elemental composition of BF, together with a decrease in cobalt and oxygen.

The EDX results, considered together with the adsorption capacities, suggest that adsorption occurs predominantly on the ZIF-67 phase, while PANI-SSA makes a comparatively smaller contribution. This behavior is consistent with the morphology and textural properties of the composites, where PANI-SSA chains only partially cover the ZIF-67 surface, leaving a significant fraction of the pore network accessible. Such a structure is plausible considering the mechanochemical synthesis route employed. The observed decrease in the detected Co content after adsorption is consistent with surface coverage by adsorbed dye molecules rather than significant changes in the composite composition, suggesting that the ZIF-67/PANI-SSA composites retain their chemical integrity during the adsorption process.

#### 2.8.6. FTIR and Raman Spectra of the Composite Before and After BF Adsorption

**Figure 10:**
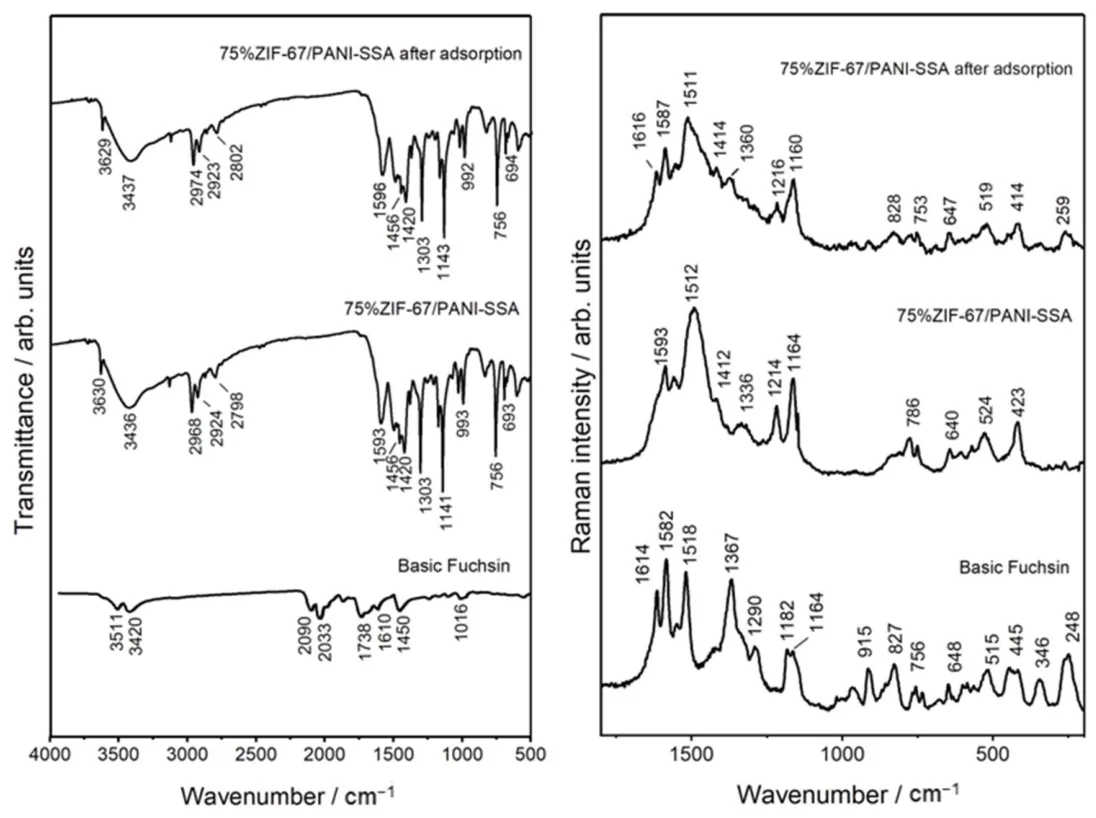
FTIR (**left**) and Raman spectra (**right**) of 75%ZIF-67-PANI-SSA composite before and after BF adsorption, as well as of pure BF dye.

To further investigate the adsorption of BF, the FTIR spectra of the 75%ZIF-67/PANI-SSA composite before and after adsorption were compared (Figure 10). The FTIR spectrum of pure BF exhibited characteristic bands at 3511 and 3420 cm^−1^, assigned to N–H stretching vibrations, together with the band at 1610 cm^−1^ related to aromatic C=C/C=N vibrations [91]. Additional bands at 1450 and 1016 cm^−1^ were attributed to aromatic C–H deformation and C–N stretching vibrations, respectively, in agreement with previously reported FTIR characteristics of BF. However, these characteristic bands of BF were not clearly distinguishable in the spectrum of the composite after adsorption. This can be attributed to the relatively low content of adsorbed dye molecules within the composite matrix and the overlapping of BF signals with the much stronger absorption bands of the adsorbent components. The FTIR spectra of the composite before and after adsorption exhibited a high degree of similarity, indicating that the adsorption process does not induce significant changes in the chemical structure of the composite. Only minor spectral variations were observed after adsorption, including a slight shift of the bands from 1593 to 1596 cm^−1^ and 1141 to 1143 cm^−1^. Furthermore, both PANI and the imidazolate linkers of ZIF-67 contain aromatic structural units with absorption bands overlapping those of BF, which may further hinder the identification of dye-related vibrations. Nevertheless, the observed spectral changes suggest interactions between BF molecules and the functional groups of the composite, while the absence of pronounced spectral changes indicates the preservation of the adsorbent structure after adsorption.

The Raman spectra recorded before and after adsorption provide clear evidence of the strong adsorption of BF (Figure 10, right). The Raman spectrum of pure BF exhibited characteristic bands related to the conjugated triphenylmethane structure. The bands at 1614 and 1582 cm^−1^ were assigned to C=C/C=N stretching vibrations of the aromatic rings, while the band at 1518 cm^−1^ was attributed to aromatic C–C stretching vibrations [92]. The bands at 1367 and 1290 cm^−1^ were related to C–N stretching and aromatic skeletal vibrations, whereas the signals at 1182 and 1164 cm^−1^ corresponded to aromatic C–H bending vibrations. The bands observed at 915, 827 and 756 cm^−1^ were assigned to out-of-plane deformation vibrations of aromatic C–H bonds. Additional bands below 900 cm^−1^ corresponded to out-of-plane aromatic C–H vibrations. Similar to the FTIR results, the characteristic Raman bands of the composite were retained after adsorption, confirming the preservation of its structural framework. Nevertheless, several subtle spectral changes were observed. The band located at 1593 cm^−1^ was shifted to 1587 cm^−1^, whereas the band at 1512 cm^−1^ remained essentially unchanged and appeared at 1511 cm^−1^ after adsorption. In addition, new bands at 1616 and 828 cm^−1^ became visible after adsorption and correspond well to the characteristic Raman vibrations of BF, indicating the presence of dye molecules on the composite surface. Furthermore, the bands originally observed at 786, 640, and 423 cm^−1^ appeared at 753, 647, and 414 cm^−1^, respectively, after adsorption, suggesting interactions between the adsorbed dye and the composite. Overall, the Raman results strongly support the successful adsorption of BF while confirming that the adsorption process does not compromise the structural stability of the 75%ZIF-67/PANI-SSA composite. Raman spectroscopy was more successful in detecting adsorbed dye molecules because it is a sub-surface technique, unlike FTIR which provides true bulk analysis.

#### 2.8.7. Evaluation of Adsorbent Reusability for BF Adsorption

**Figure 11:**
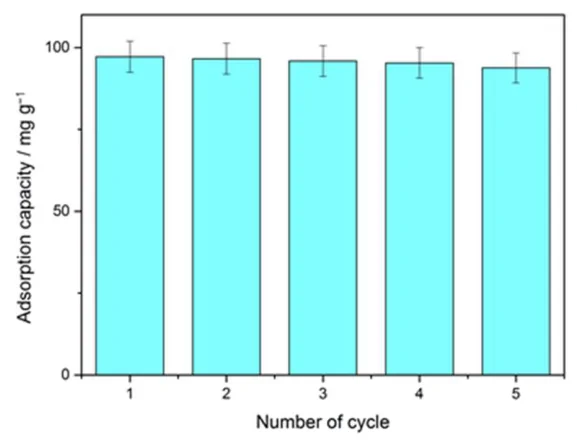
Reusability performance of the 75%ZIF-67/PANI-SSA composite over five consecutive adsorption–desorption cycles for BF removal. The concentration of adsorbent in a suspension was 1.0 g L^−1^. The initial BF concentration was 100 mg L^−1^.

The reusability of the 75%ZIF-67/PANI-SSA composite was evaluated over five consecutive adsorption–desorption cycles. Regeneration of the spent adsorbent was performed by ethanol washing, exploiting the good solubility of BF in ethanol to facilitate dye desorption. After each adsorption cycle, the regenerated adsorbent was reused under identical experimental conditions. The initial adsorption capacity of the composite toward BF was 97.2 mg g^−1^ after 6 h of adsorption. The composite maintained comparable adsorption performance after regeneration, with only a slight decrease in adsorption capacity over the five consecutive cycles. As shown in Figure 11, the adsorption capacity decreased to 93.8 mg g^−1^ after the fifth cycle, corresponding to the retention of approximately 96.5% of the initial adsorption capacity. These results demonstrate the high regeneration efficiency and excellent reusability of the 75%ZIF-67/PANI-SSA composite, indicating its potential for repeated application in BF removal from wastewater.

#### 2.8.8. Adsorption Performance of 75%ZIF-67/PANI-SSA Composite in Comparison with Other Adsorbents

To further evaluate the adsorption performance of the 75%ZIF-67/PANI-SSA composite, its adsorption behavior was compared with previously reported adsorbents for BF removal, as summarized in Table 7. A wide range of adsorbent materials, including metal oxides [93], clay-based materials [94,95], carbonaceous adsorbents [96], and polymeric materials [97], have been investigated for the removal of BF from aqueous solutions. The comparison includes the adsorption efficiency, maximum adsorption capacity (q_max_), applied adsorption isotherm model, main adsorption interactions, and the corresponding experimental conditions. As presented in Table 7, the reported adsorption capacities vary considerably depending on the physicochemical characteristics of the adsorbent as well as on experimental parameters, such as initial dye concentration, contact time, temperature, and adsorbent dosage. Therefore, direct comparison of adsorption performance among different studies should be interpreted with caution, considering the significant differences in experimental conditions. Despite these variations, electrostatic interactions were commonly recognized as the primary driving force for the adsorption of cationic BF molecules. Depending on the adsorbent composition and surface chemistry, additional adsorption mechanisms, including π–π interactions, hydrogen bonding, and ion exchange, have also been proposed. These observations highlight that the adsorption mechanism is generally governed by the synergistic contribution of several types of interactions.

Most of the reported adsorbents exhibit q_max_ values below 500 mg g^−1^ [91,97,98], while several materials show considerably lower adsorption capacities despite achieving relatively high removal efficiencies. In contrast, the 75%ZIF-67/PANI-SSA composite developed in this work exhibited a high q_max_ of 910 mg g^−1^, obtained from the Langmuir–Freundlich isotherm model, together with a removal efficiency of 97.2%, indicating its excellent adsorption performance toward BF. The superior adsorption behavior of the composite can be attributed to the synergistic contribution of both components. The porous framework of ZIF-67 provides a high density of accessible adsorption sites and facilitates dye diffusion, whereas PANI-SSA introduces aromatic structures and functional groups capable of interacting with BF molecules. Consequently, the adsorption process is governed by multiple interaction mechanisms, including electrostatic interactions between the charged surface and dye molecules and π–π interactions between aromatic rings of the adsorbent and dye molecules. The coexistence of these interactions enhances the affinity of the composite toward BF and contributes to its high adsorption capacity. Overall, the comparison presented in Table 7 demonstrates that the developed 75%ZIF-67/PANI-SSA composite exhibits competitive adsorption performance compared with previously reported materials for BF removal. This is the result of the high specific surface and the synergistic interaction between ZIF-67 and PANI-SSA, leading to the highest maximum adsorption capacity among the adsorbents investigated in this work. These results highlight the potential of the developed composite as an efficient adsorbent for the treatment of dye-contaminated wastewater.

**Table 7:** Comparison of the adsorption performance of the developed 75% ZIF-67/PANI-SSA composite with previously reported adsorbents for BF removal.

Adsorbent	Experimental Conditions	Removal Efficiency (%)	Maximum Adsorption Capacity from Isotherm Model (q_max_, mg g^−1^)	Isotherm Model	Main Adsorption Interactions	Reference
e-ZSM-5	C_0_ = 10 mg L^−1^; 2 h	99.6	251.87	Langmuir	Electrostatic, hydrogen bonding	[9]
Y_2_O_3_-doped ZnO nanoparticles	25 °C; 3 h	/	75.53	Freundlich	Electrostatic, hydrogen bonding	[93]
Natural clay of Gankawa	30 °C; 1 h	/	287	Langmuir Freundlich	Hydrogen bonding	[94]
ZrO_2_/MgMn_2_O_4_/Mg(Mg_0.333_Mn_1.333_)O_4_	C_0_ = 25 mg L^−1^; 25 °C; 3 h	75	239.81	Langmuir	Electrostatic	[99]
Local natural clay	C_0_ = 150 mg L^−1^; 6 h	99.17	149.2	Langmuir	Cation exchange, electrostatic	[95]
g-C_3_N_4_-Modified ZnO nanocomposite	C_0_ = 100 mg L^−1^; 24 h	63.69	478.14	Freundlich	Electrostatic, π–π, hydrogen bonding	[98]
MgO/BaCO_3_/BaCrO_4_/C nanocomposites	25 °C; 50 min	95.75	442.48	Langmuir	Electrostatic, π–π	[91]
Polymer–metal oxide composites	C_0_ = 25 mg L^−1^; 30 °C; 1 h	98	472	Liu Langmuir	Electrostatic, n-π^*^, hydrogen bonding	[97]
Natural bentonite	C_0_ = 100 mg L^−1^; 35 min	/	129	Langmuir	Electrostatic	[100]
Bentonite composites	C_0_ = 125 mg L^−1^; 1 h	/	131.57	Langmuir	Electrostatic, π–π, hydrogen bonding	[101]
Activated carbon derived from Lippia alba leaves	1 h	98.7	60.39	Freundlich	Electrostatic, π–π stacking	[96]
75%ZIF-67/PANI-SSA	C_0_ = 100 mg L^−1^; 25 °C; 6 h	97.2	910	Langmuir–Freundlich	Electrostatic, π–π, hydrogen bonding	Present study

## 3. Materials and Methods

### 3.1. Materials

Aniline (p.a., Centrohem, Stara Pazova, Serbia) was distilled under reduced pressure and stored under argon at room temperature, prior to use. Ammonium peroxydisulfate (APS), 5-sulfosalicylic acid (SSA) and ammonium hydroxide solution (NH_4_ OH, 25%) were used as received from Centrohem (Stara Pazova, Serbia). Cobalt nitrate hexahydrate (≥98%, Co(NO_3_)_2_·6H_2_O), 2-methylimidazole (99%, Hmim), triethylamine (TEA) and basic fuchsin (BF) were purchased from Sigma-Aldrich (St. Louis, MO, USA) and used as received.

### 3.2. Synthesis of ZIF-67

ZIF-67 was synthesized using the mixed-base (NH_4_OH/TEA) procedure developed by Li et al. [91,102], which was modified by increasing the reaction scale, extending the reaction time, and diluting the reaction mixture just before filtration, as described in our previous work [56]. In a standard synthesis procedure, 2.3284 g of cobalt nitrate hexahydrate (Co(NO_3_)_2_ · 6H_2_O) was dissolved in 12 g of deionized water and labelled as solution A. Then, 1.312 g of Hmim and 8.92 mL TEA were added to 15.04 g of ammonium hydroxide solution (25%), resulting in the solution labelled as B. Solution B was added into solution A while stirring. After 10 min, the reaction mixture was dispersed in 500 mL of deionized water and stirred for 30 min. The resulting precipitate was collected by filtering and rinsed with deionized water until achieving a pH value of ~7. Final purple powder ZIF-67 was obtained by drying in a vacuum at 60 °C for 4 h.

### 3.3. Synthesis of PANI-SSA

PANI-SSA was synthesized through the oxidative chemical polymerization of aniline monomer in aqueous solution of SSA, utilizing APS as the oxidizing agent and mole ratio [SSA]/[aniline] = 0.25, chosen to obtain 1-D nanostructured PANI, according to ref. [46]. Initially, 2 mL (2.2 × 10^–2^ mol) of aniline was dissolved in 190 mL of aqueous solution containing 1.4 g (5.5 × 10^–3^ mol) of SSA. The solution was heated to boiling and then cooled to room temperature. Then, 100 mL of the oxidant ammonium persulfate (0.22 M in water) was added into the above solution at ~20 °C to start the oxidation, and the reaction mixture was stirred for 3 h. The precipitated PANI-SSA was collected on a filter after 180 min of reaction, rinsed three times with 5 × 10^–3^ M SSA, and dried in a vacuum at 60 °C for 3 h.

### 3.4. Preparation of ZIF-67/PANI-SSA Composites

ZIF-67/PANI-SSA composites containing different weight fractions of ZIF-67 (20, 25, 50 and 75 wt.%) were prepared by mechanochemical mixing method (mortar–pestle liquid-assisted manual grinding) of pre-synthesized PANI-SSA and ZIF-67. The total mass of the prepared composites was maintained at 500 mg. The amounts of ZIF-67 and PANI-SSA used for the preparation of composites containing 20, 25, 50 and 75 wt.% ZIF-67 were 100/400 mg, 125/375 mg, 250/250 mg and 375/125 mg, respectively. The entire amount of precursors was entrapped in the synthesized composite, without losses. To prepare the composite, measured amounts of PANI-SSA and ZIF-67 were placed in a mortar with a small amount (1 mL) of ethanol, crushed, mashed and mixed (grinded) with a pestle until the solvent completely evaporated (c.a. 10 min). The resulting composite was then air-dried at room temperature and then in a vacuum at 60 °C for 3 h. The composite samples are labeled as ZIF-67/PANI-SSA with the prefix 20%, 25%, 50% and 75% according to the weight fraction of ZIF-67.

### 3.5. Characterization Techniques

Fourier-transform infrared (FTIR) spectra of the samples were recorded using a Nicolet iS20 spectrometer (Thermo Fisher Scientific, Waltham, MA, USA) with the KBr pellet technique. Spectra were collected in the range of 4000–400 cm^−1^, with a resolution of 2 cm^−1^ and 32 scans per spectrum.

Raman spectra were recorded using a DXR Raman Microscope (Thermo Fisher Scientific, Waltham, MA, USA) with excitation provided by a 532 nm diode-pumped solid-state laser. The laser beam was focused on a 2.1 μm spot on the sample surface through a 10× microscope objective. The acquisition time was 10 s with 10 accumulations per spectrum. The scattered light was analyzed using a spectrograph equipped with a 50 μm pinhole aperture and a 900 lines mm^−1^ grating.

Thermogravimetric analysis (TGA) was performed using an SDT 2960 thermoanalytical device (TA Instruments, New Castle, DE, USA) to examine the thermal properties of pristine ZIF-67, PANI-SSA, and their composites. Samples were heated from ambient temperature to 800 °C at a rate of 10 °C min^−1^ under a synthetic air flow of 80 mL min^−1^. The degradation temperature of each sample was determined based on the corresponding TGA curves.

The cobalt content in the synthesized materials was quantified by flame atomic absorption spectrometry (FAAS) following microwave-assisted acid digestion. Prior to measurement, the samples were completely dissolved using an ETHOS 1 Advanced Microwave Digestion System (Milestone, Bergamo, Italy). Precisely weighed portions of the composites were treated with a mixture of concentrated nitric (2 mL, 65%) and hydrochloric acid (6 mL, 36%) in Teflon vessels. The closed vessels were heated under controlled conditions, first to 160 °C within 8 min and then to 210 °C over the next 5 min, maintaining this temperature for an additional 20 min. After cooling to room temperature, the digested solutions were diluted to a final volume of 50 mL with deionized water. FAAS measurements were performed using a PerkinElmer AAnalyst 700 spectrometer (Shelton, CT, USA) equipped with a cobalt hollow-cathode lamp. Calibration was carried out using a series of standard cobalt solutions of known concentrations, prepared by successive dilution of a 1000 mg/L Co stock solution (CPA Chem, Bogomilovo, Stara Zagora, Bulgaria). The cobalt concentrations in the samples were then determined from the calibration curve within the linear range of the instrument.

The morphology of the samples was analyzed using a scanning electron microscope (SEM), model JEOL JSM-6610LV (Akishima, Tokyo, Japan). Before recording micrographs, the samples were sputter-coated with a thin layer of carbon using the SCD005 device (Leica Mikrosysteme, Vienna, Austria). Surface composition of materials was probed on a PhenomProX SEM (Phenom, Eindhoven, The Netherlands) equipped with an energy-dispersive X-ray spectroscope (EDX).

The electrical conductivity of the samples was measured at room temperature using an LCR meter (model LCR-6100, GW Instek, New Taipei City, Taiwan) at a fixed frequency of 1.0 kHz. The samples were compressed into pellets between two stainless steel pistons, and a constant pressure of 255 MPa was applied using a manual hydraulic press during the measurements.

Textural parameters, including specific surface area (S_BET_), total pore volume (V_tot_), and pore size distribution (PSD), were determined from nitrogen adsorption isotherms at −196.15 °C using a BELSORP-MINI X gas sorption analyzer (MicrotracBEL, Osaka, Japan). Before the analysis, the samples were degassed under vacuum at 195 °C for 24 h to eliminate residual adsorbates on the surface and ensure reliable measurements. The degassing temperature was selected based on the results of the TGA analysis. The BET analysis, in combination with the Rouquerol method [103], was used to estimate surface areas (S_BET_) and total pore volume (V_tot_). Further analyses of the obtained N_2_ adsorption isotherms were done using the MP plot method to analyze micropores [104] and the Barrett–Joyner–Halenda (BJH) method to analyze the mesoporous domain [105].

For zeta-potential measurements, a suspension of the 75%ZIF-67/PANI-SSA sample was prepared by dispersing 1 mg of the composite in 10 mL of 10 mM KCl solution. The suspension was sonicated in an ultrasonic bath for 10 min to ensure adequate particle dispersion. The electrophoretic zeta potential was then determined using a Zetasizer Nano ZS90 instrument (Malvern Instruments, Worcestershire, UK). Prior to the measurements, the instrument was calibrated using the manufacturer’s standard latex dispersion with a certified zeta-potential value of −50 ± 5 mV.

### 3.6. Adsorption Studies

Adsorption experiments were carried out in batch systems to achieve the optimum operating conditions for the removal of BF from aqueous solutions using ZIF-67, PANI-SSA, and four ZIF-67/PANI-SSA composite samples. A calibration curve for BF dye was constructed using a UV-Vis spectrophotometer (Evolution 220, Thermo Scientific, Waltham, MA, USA) by measuring the absorbance of standard solutions in a range of known concentrations (0.5–50 ppm) of dye solution at λ_max_ = 546 nm. A linear relationship was obtained and used to determine the unknown concentrations of the dye in the treated solutions. The suspension used in the tests comprised 1 mg/mL of the adsorbent and BF solutions with concentrations ranging from 20 to 1000 mg L^−1^. This concentration range was selected to reflect the high dye levels reported in textile industrial effluents and to evaluate adsorption performance under severe contamination conditions [95]. The experiment was carried out on a thermostatic laboratory shaker OLS Shaking Bath (Grant Instruments, Royston, UK) at 110 rpm and 25 °C. After 5–300 min, the dispersion was centrifuged at 4000 rpm for 5 min to remove the adsorbents from the solution. The residual dye concentration in the supernatant was determined using a UV–Vis spectrophotometer (Evolution 220, Thermo Scientific, Waltham, MA, USA).

The adsorption capacity (*q_t_*) at any time, *t*, and the removal efficiency (%*R*) were calculated using the following equations:

$$q_{t}=\frac{\left(C_{0}-C_{t}\right)V}{m}$$

$$\%R=\frac{\left(C_{0}-C_{t}\right)∙100\%}{C_{0}}$$

where *C*_0_ and *C_t_* (mg L^−1^) are the initial and time-dependent dye concentrations, *V* (L) is the solution volume, and *m* (g) is the mass of the adsorbent. At equilibrium, *C_t_* = *C_e_* and *q_t_* = *q_e_.* The impact of the contact time was evaluated by varying the contact time (0 to 360 min) at room temperature.

#### 3.6.1. Kinetics of Adsorption and Adsorption Isotherms

Equilibrium adsorption isotherms are critical for understanding the interaction between the adsorbate and adsorbent. In this study, the Langmuir, Freundlich and Langmuir–Freundlich models were used to analyze the adsorption affinity of the adsorbents toward BF molecules. The Langmuir isotherm, assuming monolayer adsorption on a homogeneous surface, is described as follows [106]:

$$\frac{C_{e}}{q_{e}}\;=\frac{1\;}{K_{L}q_{m}}\;+\;\frac{C_{e}\;}{q_{m}}$$

where *q_m_* is the maximum capacity of the adsorbent (mg g^−1^), *C_e_* is the adsorbate concentration in solution at equilibrium (mg L^−1^), and *K_L_* is the adsorption constant related to the binding sites affinity (L mg^−1^). Values of *q_m_* and *K_L_* were determined from the slope and intercept of a plot of *C_e_/q_e_* versus *C_e_*.

The Freundlich isotherm, assuming a heterogeneous surface with a non-uniform distribution of adsorption energies, is expressed as follows:

$$log\;q_{e}=\;logK_{F}\;+\frac{\;logC_{e}}{n}$$

where *K_F_* is the Freundlich isotherm constant (L g^−1^), an approximate indicator of adsorption capacity, while *n* is related to adsorption intensity, with 1/*n* indicating the strength of adsorption [107]. The values of *K_F_* and *n* are determined from the intercept and slope, respectively, of the plot of *logq_e_* versus *logC_e_* in the Freundlich equation.

The Langmuir–Freundlich isotherm was proposed to describe adsorption on heterogeneous surfaces [108]. It illustrates how adsorption sites are distributed across the adsorbent surface. At low adsorbate concentrations, the model behaves as the Freundlich isotherm, while at high concentrations, it approaches the Langmuir isotherm. The Langmuir–Freundlich equation is expressed as follows:

$$q=q_{max}\;\frac{K∙C^{\frac{1}{n}}}{1+K∙C^{\frac{1}{n}}}$$

where *q_max_* represents the maximum adsorption capacity (mg g^−1^), *K* is the equilibrium constant associated with the heterogeneous surface, and 1/*n* is the heterogeneity parameter ranging from 0 to 1. The kinetic study was performed to better understand the adsorption rate, reaction order, and the time required to reach equilibrium. That study suggested that the adsorption equilibrium was attained within about 180 min. To describe the adsorption behavior of BF on ZIF-67, PANI-SSA, and the ZIF-67/PANI-SSA composite samples, two widely used kinetic models, the pseudo-first-order model and pseudo-second-order model, were applied using linear regression analysis.

The pseudo-first-order model, described by the Lagergren equation [109], is expressed as follows:

$$q_{t}\;=\;q_{e}(1\;-\;e^{-k_{1}t})$$

where *q_t_* and *q_e_* (mg g^−1^) are the adsorption capacities at time *t* (min) and at equilibrium, respectively, and *k_1_* (min^−1^) is the rate constant of pseudo-first-order adsorption. A plot of log (*q_e_* − *q_t_*) versus *t* gives a straight line from which *k*_1_ and *q_e_* can be determined from the slope and intercept, respectively.

The pseudo-second-order model, described by Ho et al. [110], is given as follows:

$$\frac{t}{q_{t}}\;=\;\frac{1}{k_{2}q_{e}^{2}}\;+\frac{t}{q_{e}}$$

where *k*_2_ (g mg^−1^ min^−1^) is the pseudo-second-order rate constant, and *q_e_* is the theoretical adsorption capacity at equilibrium. The initial adsorption rate, *h*, can be calculated using *h = k*_2_*q_e_*^2^. A plot of *t*/*q_t_* versus *t* allows determination of *k_2_* and *q_e_* from the intercept and slope, respectively.

#### 3.6.2. Adsorbent Reusability Test

The reusability of the 75%ZIF-67/PANI-SSA composite for BF adsorption was evaluated over five consecutive adsorption–desorption cycles. After each adsorption experiment, the spent adsorbent was regenerated using ethanol, in which BF is soluble. Briefly, 50 mg of the spent composite was dispersed in 5 mL of ethanol and treated in a thermostatic laboratory shaker (OLS Shaking Bath, Grant Instruments, Royston, UK) at 110 rpm and 25 °C for 12 h. The regenerated material was then separated by centrifugation, washed three times with ethanol, air-dried at room temperature, and subsequently dried under vacuum at 60 °C for 3 h. The regenerated adsorbent was reused in subsequent adsorption experiments performed at 25 °C to evaluate its adsorption performance after regeneration.

## 4. Conclusions

In this study, a series of ZIF-67/PANI-SSA composites with different contents of the two components (20, 25, 50 and 75 wt.% of ZIF-67) was synthesized through a simple and environmentally friendly mechanochemical method, with nanostructured PANI-SSA pre-synthesized via chemical oxidative polymerization of aniline with SSA as a dopant. Comprehensive physicochemical characterization confirmed successful integration of ZIF-67 and PANI-SSA into composites and revealed strong interfacial interactions between the two components. FTIR and Raman spectroscopies demonstrated that the structural integrity of both components was largely preserved in the composites, with partial dedoping of PANI due to its interaction with ZIF-67. SEM analysis showed significant changes in morphology with changing weight ratio of components, with 1-D nanostructures of PANI-SSA observable for composites with 20 wt.% and 25 wt.% ZIF-67 and granular morphology as dominating for composites with a higher content of ZIF-67. TGA revealed the improved thermal stability of all composites compared to individual components. It is manifested in slower weight loss for composites in the temperature range 235−425 °C compared to pure ZIF-67 and PANI-SSA and in higher temperatures of complete decomposition/transformation to Co_3_O_4_ (450, 460, 500 and 550 °C for composites synthesized with 75, 50, 25 and 20 wt.% of ZIF-67), compared to pure ZIF-67 (295 °C), reflecting the synergistic action of PANI-SSA and ZIF-67. The temperature of complete composite decomposition increases with increasing PANI content, meaning that polymer chains have a protective effect in terms of ZIF-67 thermal degradation. The electrical conductivity of composites (10^−5^–10^−6^ S cm^−1^) is between that of pure ZIF-67 (10^−8^ S cm^−1^) and PANI (10^−2^ S cm^−1^) and decreases with increasing ZIF-67 fraction due to its insulating nature. Adsorption experiments demonstrated that all composites are better adsorbents for BF dye compared to PANI-SSA, with the 75%ZIF-67/PANI-SSA composite exhibiting the highest values of maximum adsorption capacity (910 mg g^−1^) and removal efficiency (97.2%), which even exceed those of pure ZIF-67. The adsorption process follows pseudo-second-order kinetics, fitting well to the Langmuir–Freundlich isotherm, which indicates favorable monolayer adsorption on a heterogeneous surface. Electrostatic and π–π interactions, as well as hydrogen bonds, are proposed to be key driving forces for BF adsorption on the composites. The outstanding adsorption performance of 75%ZIF-67/PANI-SSA arises from the preservation of substantial ZIF-67 porosity and specific surface area together with positive interfacial synergy introduced by PANI-SSA. This combination provides both a high number of accessible adsorption sites and efficient utilization of the composite surface. It was shown that the composite adsorbent retains its structural integrity and can be efficiently regenerated via a simple ethanol-washing treatment after BF adsorption, with a highly preserved adsorption capacity after multiple regeneration cycles. The synergistic combination of the high surface area and porosity of ZIF-67 with the functional groups and counter-ions of PANI-SSA, which modify the surface charge and provide new active sites, resulted in a composite material with enhanced physicochemical properties (thermal stability, electrical conductivity) and superior adsorption performance compared to pure ZIF-67 and PANI-SSA. These findings highlight the great potential of ZIF and PANI composites as multifunctional adsorbents for efficient dye removal and broader environmental remediation applications.

## Figures and Tables

**Table 1:** Electrical conductivity and cobalt content determined by FAAS for PANI-SSA, ZIF-67 and ZIF-67/PANI-SSA composites.

Sample	Conductivity (S cm^−1^)	Co Content (wt.%)
PANI-SSA	3.4 · 10^−2^	–
ZIF-67	2.9 · 10^−8^	23.2
20%ZIF-67/PANI-SSA	1.3 · 10^−5^	4.7
25%ZIF-67/PANI-SSA	8.4 · 10^−6^	6.3
50%ZIF-67/PANI-SSA	3.1 · 10^−6^	12.9
75%ZIF-67/PANI-SSA	1.6 · 10^−6^	22.0

**Table 2:** Weight loss, weight residue and temperature of complete degradation of ZIF-67, PANI-SSA and ZIF-67/PANI-SSA composites, determined by TGA.

Sample	Weight Loss (wt.%) 25–200 °C/200–800 °C (H_2_O Content)	Residue at 800 °C (wt.%)	T of Complete Degradation (°C)
PANI-SSA	2.8/58.0	39.2	295
ZIF-67	6.7/92.5	0.8	625
20%ZIF-67/PANI-SSA	5.6/87.3	7.1	545
25%ZIF-67/PANI-SSA	5.1/86.2	8.7	495
50%ZIF-67/PANI-SSA	4.8/78.3	16.9	460
75%ZIF-67/PANI-SSA	4.4/63.2	32.4	450

**Table 3:** Textural properties of pure PANI-SSA, pure ZIF-67, and ZIF-67/PANI-SSA composites. The MP method was used as the principal indicator of microporosity. The surface areas and pore volumes obtained by the BET, MP, and BJH methods are model-dependent and overlapping quantities and were therefore not treated as additive. In Table 3, C is the dimensionless BET constant, R^2^ is the coefficient of determination, S_p_ and V_p_ are the method-derived pore surface area and pore volume, respectively, and d_av_ is the BJH average pore diameter.

	ZIF-67	PANI-SSA	75%ZIF-67/ PANI-SSA	50%ZIF-67/ PANI-SSA	25%ZIF-67/ PANI-SSA	20%ZIF-67/ PANI-SSA
BET analysis						
S_BET_/m^2^ g^−1^	841	24.9	651	228	11.0	13.7
V_tot_/cm^3^ g^−1^	0.440	0.088	0.310	0.139	0.041	0.047
C	1138	68.4	362	436	80.6	71.8
R^2^	0.9993	0.9999	0.9999	0.9999	0.9999	0.9999
MP analysis (pore diameter < 2.7 nm)						
S_p_/m^2^ g^−1^	966	/	713	262	/	/
V_p_/cm^3^ g^−1^	0.323	/	0.259	0.080	/	/
BJH analysis (pore diameter > 1 nm)						
S_p_/m^2^ g^−1^	757	38.1	583	182	22.5	29.8
V_p_/cm^3^ g^−1^	0.317	0.081	0.230	0.096	0.032	0.046
d_av_/nm	1.677	8.517	1.577	2.107	5.738	6.198

**Table 4:** Pseudo-second-order kinetics parameters for the adsorption of BF on ZIF-67, PANI-SSA and ZIF-67/PANI-SSA composites.

	ZIF-67	PANI-SSA	75%ZIF-67/PANI-SSA	50%ZIF-67/PANI-SSA	25%ZIF-67/PANI-SSA	20%ZIF-67/PANI-SSA
q_e_/mg g^−1^	97.3 ± 0.2	34.8 ± 0.9	97.9 ± 0.2	93.5 ± 0.2	92.9 ± 0.5	91.8 ± 0.4
k_2_/g mg^−1^ min^−1^	0.011 ± 0.003	0.0017 ± 0.0004	0.004 ± 0.002	0.00036 ± 0.0003	0.0014 ± 0.0002	0.0013 ± 0.0002
h/mg g^−1^ min^−1^	100 ± 20	2.1 ± 0.6	33 ± 12	31 ± 3	12 ± 2	11 ± 2
R^2^	0.99999	0.99934	0.99999	0.99999	0.99984	0.99986
Adj. R^2^	0.99999	0.99927	0.99999	0.99999	0.99982	0.99984

**Table 5:** Adsorption parameters based on the Langmuir–Freundlich isotherm.

	ZIF-67	PANI-SSA	75%ZIF-67/PANI-SSA	50%ZIF-67/PANI-SSA	25%ZIF-67/PANI-SSA	20%ZIF-67/PANI-SSA
q_max_/mg g^−1^	900 ± 40	160 ± 20	910 ± 30	820 ± 30	670 ± 30	510 ± 30
K/L^n^ mg^−n^	0.029 ± 0.004	0.001 ± 0.001	0.027 ± 0.003	0.017 ± 0.005	0.021 ± 0.004	0.019 ± 0.002
1/n	0.9 ± 0.1	1.1 ± 0.3	0.9 ± 0.1	0.9 ± 0.2	0.9 ± 0.2	0.9 ± 0.1
R^2^	0.9987	0.9975	0.9988	0.9885	0.9960	0.9993
Adj. R^2^	0.9974	0.9970	0.9976	0.9871	0.9921	0.9992

**Table 6:** Elemental compositions of ZIF-67, 25%ZIF-67/PANI-SSA, and 75%ZIF-67/PANI-SSA before and after adsorption of BF, determined by EDX mapping.

Sample	State	Content (at.%)			
		C	N	O	Co
ZIF-67	Before adsorption	33.4	24.7	33.6	8.3
	After adsorption	50.8	34.0	11.8	3.4
75%ZIF-67/PANI-SSA	Before adsorption	47.9	24.3	19.9	7.9
	After adsorption	52.3	30.7	10.4	6.6
20%ZIF-67/PANI-SSA	Before adsorption	57.3	22.5	18.9	1.3
	After adsorption	64.3	24.2	11.0	0.5

## Data Availability

The data presented in this study are available on request from the corresponding author.
